# Supplementary material for: Single-Exciton Photoluminescence in a GaN Monolayer inside an AlN Nanocolumn
Source: Nanomaterials (Basel). 2023 Jul 12;13(14):2053. doi: 10.3390/nano13142053 (PMC10386294; doi:10.3390/nano13142053)
Supplement: Supplementary file 1 [file nanomaterials-13-02053-s001.zip › nanomaterials-2452760-supplementary.pdf]

nanomaterials

Supplementary

Single-exciton photoluminescence in a GaN monolayer inside an AlN nanocolumn

Eugenii Evropeitsev, Dmitrii Nechaev, Valentin Jmerik, Yuriy Zadiranov, Marina Kulagina, Sergey Troshkov, Yulia Guseva, Daryia Berezina, Tatiana Shubina, and Alexey Toropov*

Ioffe Institute, 26 Politekhnicheskaya, 194021 St. Petersburg, Russia; john_fzf@mail.ru (E.E.); nechayev@mail.ioffe.ru (D.N); jmerik@pls.ioffe.ru (V.J.); zadiranov@mail.ioffe.ru (Y.Z.); marina.kulagina@mail.ioffe.ru (M.K.); S.Troshkov@mail.ioffe.ru (S.T.); Guseva.Julia@mail.ioffe.ru (Y.G.); Dariya.Burenina@mail.ioffe.ru (D.B.); shubina@beam.ioffe.ru (T.S.); toropov@beam.ioffe.ru (A.T.)
* Correspondence: toropov@beam.ioffe.ru

Figure S1 shows a schematic of the experimental setup used to measure the spectra and decay curves of photoluminescence. The sample was fixed in a He-flow microcryostat (ST-500, Janis). The PL was excited by the fourth harmonic of a mode locked Ti-sapphire laser (Mira-900 with a harmonics generator, Coherent), operating in a pulsed mode with a pulse repetition period of 13 ns and a pulse duration of 120 fs, pumped by the light of a continuous wave Nd:YAG laser (Verdi V10, Coherent). The light of the fourth harmonic (λ=215 nm), reflected from the long-pass interference filter (RazorEdge 224 nm, Semrock), was focused onto the sample, using a 40x reflex objective lens (LMM-40X-UVV, Thorlabs). The size of the excitation spot was ~ 10 µm. PL radiation collected by the same objective lens was focused by a fluorite lens (f=18 cm) to a mirror diaphragm (pinhole) with the diameter of 200 µm, located in the plane of the enlarged image of the sample. With the illuminator turned on, the image of the sample surface could be observed using a USB-camera. To select the required region of PL detection, the reflex objective lens was positioned using a XYZ linear translator with a minimal step of about 30 nm. Radiation transmitted through the pinhole was guided to a spectrometer (Acton SP2500, Princeton Instruments) with a diffraction grating of 1800 grooves/mm. The PL decay curves at a selected spectral band and PL spectra were detected by a single-photon photomultiplier (PMC-100-4, Becker & Hickl) and a cooled CCD array (Pylon, Princeton Instruments), respectively. The signal from a single-photon photomultiplier was applied to a time-correlated single photon counting module (SPC-130, Becker & Hickl). A pin photodiode (PHD-400, Becker & Hickl) was used for synchronization with the pulses of the fundamental harmonics of the Ti-sapphire laser. Measured spectra and decay curves were normalized to the excitation power registered during the measurements by a power meter (11PD100-SiUv, Standa).

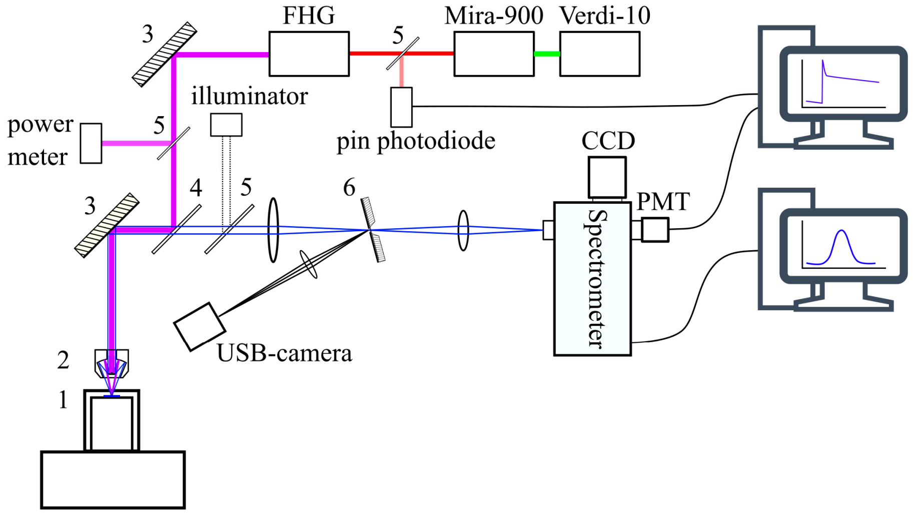

Figure S1. Schematic diagram of the experimental micro-PL setup: 1 – microcryostat, 2 – reflective objective, 3 – aluminum UV-enhanced mirror, 4 – long-pass interference filter (cutoff at a wavelength of 224 nm), 5 – fluorite plate, 6 – pinhole, FHG – forth harmonic generator.

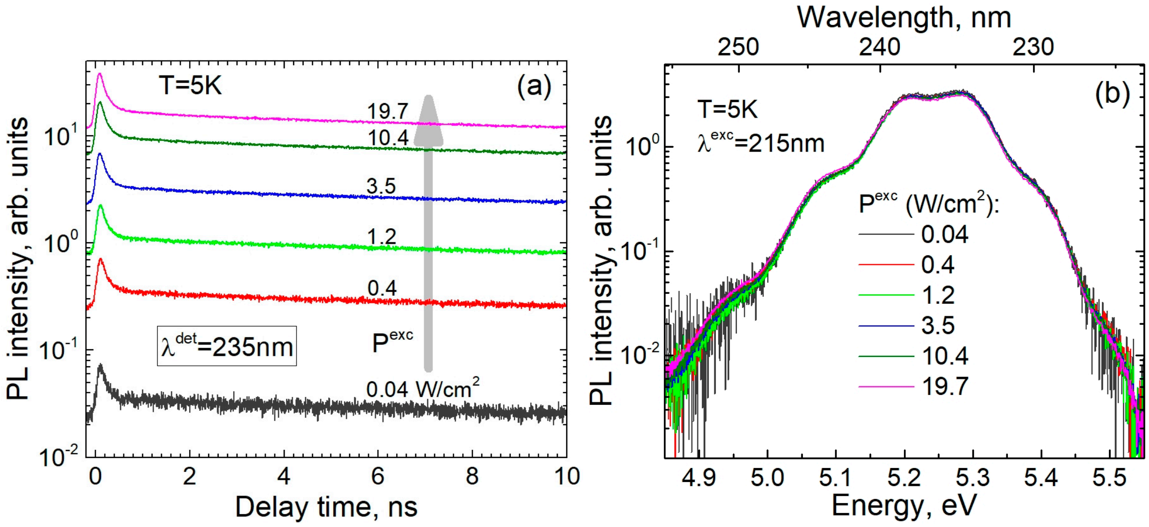

Figure S2. (a) Time-resolved decay curves for a low-temperature PL of a planar 1.1 ML thick GaN/AlN QW, obtained with the excitation power varied from 0.04 to ~20 W/cm². The excitation and detection wavelengths are 215 and 235 nm, respectively. **(b)** Corresponding time-integrated PL spectra normalized to the excitation power.

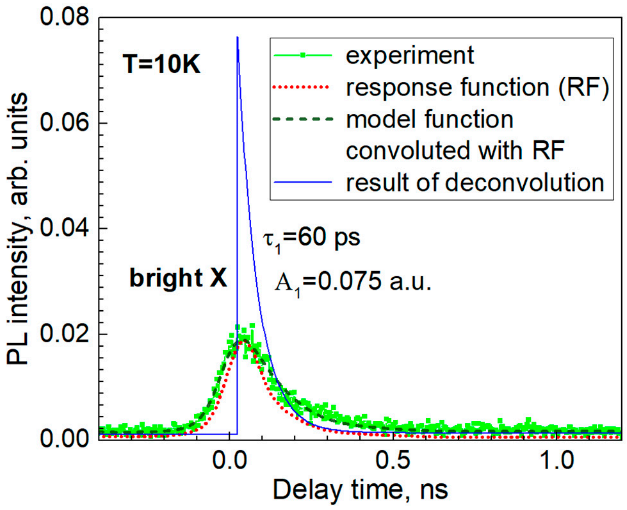

Figure S3. Results of approximation of the PL decay curve of a localized bright exciton. The dotted line is the instrumental response function (RF), the dashed line is the model function with optimal fitting parameters, convoluted with the RF, the solid line – is the result of deconvolution, i.e. the original model function with optimal values of the fitting parameters.
